# Do carotid MR surface coils affect PET quantification in PET/MR imaging?

**DOI:** 10.1186/2197-7364-2-S1-A34

**Published:** 2015-05-18

**Authors:** Martin J Willemink, Mootaz Eldib, Tim Leiner, Zahi A Fayad, Venkatesh Mani

**Affiliations:** Translational and Molecular Imaging Institute, Icahn School of Medicine at Mount Sinai, New York, NY USA; Department of Radiology, University Medical Center Utrecht, Utrecht, The Netherlands

To evaluate the effect of surface coils for carotid MR imaging on PET quantification in a clinical simultaneous whole-body PET/MR scanner. A cylindrical phantom was filled with a homogeneous 2L water-FDG mixture at a starting dose of 301.2MBq. Clinical PET/MR and PET/CT systems were used to acquire PET-data without a coil (reference standard) and with two carotid MRI coils (Siemens Special Purpose 8-Channel and Machnet 4-Channel Phased Array). PET-signal attenuation was evaluated with Osirix using 51 (PET/MR) and 37 (PET/CT) circular ROIs. Mean and maximum standardized uptake values (SUVs) were quantified for each ROI. Furthermore, SUVs of PET/MR and PET/CT were compared. For validation, a patient was scanned with an injected dose of 407.7MBq on both a PET/CT and a PET/MR system without a coil and with both coils. PET/MR underestimations were -2.2% (Siemens) and -7.8% (Machnet) for SUVmean, and -1.2% (Siemens) and -3.3% (Machnet) for SUVmax, respectively. For PET/CT, underestimations were -1.3% (Siemens) and -1.4% (Machnet) for SUVmean and -0.5% (both Siemens and Machnet) for SUVmax, respectively using no coil data as reference. Except for PET/CT SUVmax values all differences were significant. SUVs differed significantly between PET/MR and PET/CT with SUVmean values of 0.51-0.55 for PET/MR and 0.68-0.69 for PET/CT, respectively. The patient examination showed that median SUVmean values measured in the carotid arteries decreased from 0.97 without a coil to 0.96 (Siemens) and 0.88 (Machnet). Carotid surface coils do affect attenuation correction in both PET/MR and PET/CT imaging. Furthermore, SUVs differed significantly between PET/MR and PET/CT.

